# Ruptured Arteriovenous Malformation Presenting with Kernohan's Notch

**DOI:** 10.1155/2015/921930

**Published:** 2015-04-27

**Authors:** Christopher F. Dibble, Michael P. Wemhoff, Tarik Ibrahim, Deanna Sasaki-Adams, Sten Solander, Anand V. Germanwala

**Affiliations:** ^1^Department of Neurological Surgery, University of North Carolina School of Medicine, Chapel Hill, NC 27514, USA; ^2^Department of Neurological Surgery, Loyola University School of Medicine, Maywood, IL 60153, USA; ^3^Department of Radiology, University of North Carolina School of Medicine, Chapel Hill, NC 27514, USA; ^4^Edward Hines, Jr. VA Medical Center, Hines, IL 60141, USA

## Abstract

AVMs are congenital lesions that predispose patients to intracranial hemorrhage and resultant neurological deficits. These deficits are often focal and due to the presence of local neurologic disruption from hemorrhage in the contralateral cerebral hemisphere. We present a rare case of a patient with ipsilateral neurological deficits due to Kernohan's Notch phenomenon resulting from hemorrhage from an AVM. A 31-year-old woman with seizures underwent MR and angiographic imaging which confirmed an unruptured left parietal AVM. The patient declined treatment and presented with obtundation 4 years later. Imaging revealed an acute left parietal ICH and SDH with significant mass effect. The patient underwent emergent hemicraniectomy and hematoma evacuation. Postoperatively, she made significant improvement and was following commands contralaterally with ipsilateral hemiplegia. MR imaging revealed right Kernohan's Notch. The patient had significant rehabilitation with neurological improvement. She eventually underwent elective embolization followed by subsequent surgical resection and bone replacement. Three years from the initial hemorrhage, the patient had only mild left-sided weakness and ambulates without assistance. A false localizing sign, Kernohan's Notch phenomenon, should be considered in the setting of AVM hemorrhage with paradoxical motor impairment and can be identified through MRI.

## 1. Introduction

Kernohan's Notch refers to compression and injury of the cerebral peduncle against the tentorial edge by a contralateral mass. This phenomenon consists of the paradoxical findings of ipsilateral hemiplegia and, less frequently, ipsilateral oculomotor nerve palsy. More common in the setting of an underlying neoplasm or with trauma, this finding is rarely seen with spontaneous intracranial hemorrhages. We present a case report with long-term follow-up of a patient with a known AVM that later ruptured causing ipsilateral neurological deficits due to Kernohan's Notch.

## 2. Case Report

**Figure 1:**
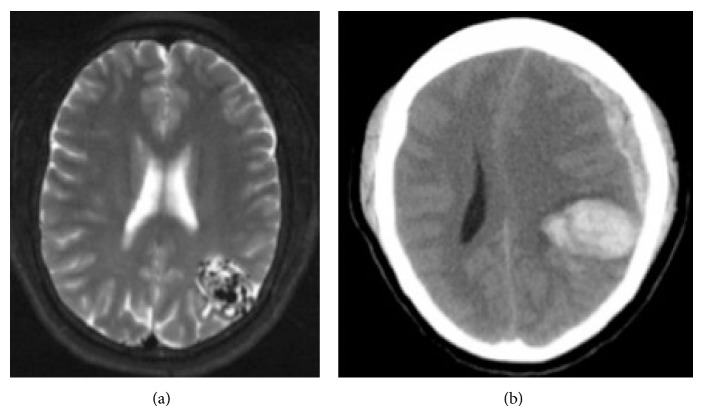
(a) Axial T2 MRI demonstrating left parietal AVM extending from the posterior horn of the left lateral ventricle to the cortex of the left parietal lobe; (b) axial CT demonstrating left parietal ICH and frontoparietal SDH. There is approximately 1.2 cm of left-to-right midline shift.

A 31-year-old woman with a normal exam presented to a tertiary care medical center with a seven-year history of seizures that were controlled with phenytoin. An MRI was obtained that revealed a left parietal AVM without any signs of hemorrhage (Figure 1(a)). An angiogram confirmed a Spetzler-Martin Grade II AVM with venous drainage into the sagittal and left transverse sinuses. Microsurgical resection was recommended but the patient declined intervention.

**Figure 2:**
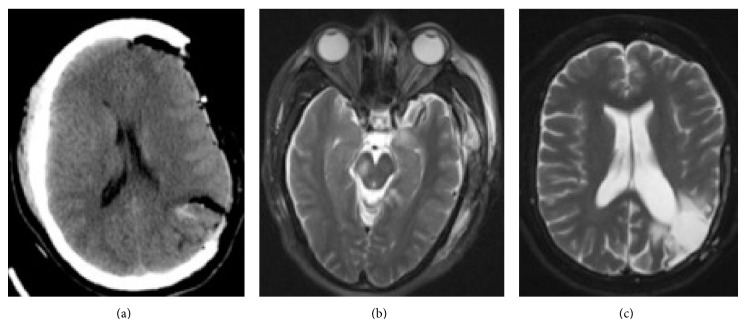
(a) Axial CT status after craniectomy, demonstrating partial clot evacuation and improved midline shift. The AVM is still present with residual hemorrhage; (b) axial T2 MRI postoperative day 2 demonstrating ischemic damage in the left hippocampus and right cerebral peduncle in the area of the corticospinal tract (Kernohan's Notch); (c) axial T2 MRI demonstrating complete resection of the AVM and cranioplasty.

Four years later, the patient was found at home acutely obtunded. She was taken to an outside hospital where her exam revealed an enlarged, nonreactive left pupil and minimal movement of the extremities. A CT scan demonstrated a left parietal ICH and SDH consistent with AVM rupture and left-to-right shift (Figure 1(b)). She was transferred to our institution and underwent an emergent decompressive hemicraniectomy with partial clot evacuation. AVM resection was not attempted at the time due to the emergent nature of the surgery. Postoperative CT confirmed successful hematoma evacuation and resolving midline shift (Figure 2(a)). Postoperatively, she was noted to have equal pupils, localization with the right arm and spontaneous movement of the right leg, a paradoxical left facial palsy, and left hemiplegia. An MRI was ordered and areas of ischemic damage were noted in the right cerebral peduncle consistent with sequelae of Kernohan's Notch phenomenon (Figure 2(b)). The patient was transferred to the rehabilitation unit for two months. Her motor exam improved to 4/5 strength in the left arm and leg; the patient was able to ambulate with the assistance of a walker.

Seven months later, she underwent partial Onyx embolization of the AVM, followed two days later by resection and cranioplasty. Postresection imaging showed no residual AVM (Figure 2(c)). Two years after resection, the patient's exam demonstrated 4+/5 strength in left upper and lower extremities, minimal facial asymmetry, and smooth, unassisted ambulation.

## 3. Discussion

AVMs display an annual average hemorrhage rate of 2.8–4.6% [1, 2]. Based on this data, at the time of initial presentation, this patient had an estimated 74% chance of hemorrhage from this AVM within her remaining lifetime. Surgical resection was initially recommended because of its high rate of success with low grade AVMs and the high risk of AVM rupture [3].

Rare Kernohan's Notch phenomenon occurs when the herniated mesial temporal lobe pushes the midbrain into the contralateral tentorial edge [4]. The rigidity of this dural structure carves out the eponymous notch and causes interruption of the fibers of the contralateral cerebral peduncle. The resulting corticospinal tract damage can manifest as an ipsilateral hemiparesis. If herniation continues without intervention to treat the mass lesion, anteroposterior elongation of the midbrain can cause compression and tearing of the paramedian perforating vessels that supply the midbrain tegmentum, resulting in so-called Duret hemorrhages, a poor prognostic indicator.

Good neurologic recovery has been reported on patients treated quickly with mass lesions resulting in Kernohan's phenomenon [5]. Despite our patient having spent an unknown amount of time being obtunded, patients with such lesions should be treated emergently in this setting to minimize midbrain compression time and maximize potential neurological recovery.

## 4. Conclusion

Kernohan's phenomenon secondary to ICH from AVM rupture is a rare and dangerous condition. Although acute neurological injury from this phenomenon can be significant, good long-term recovery can be obtained. A false localizing sign, Kernohan's Notch phenomenon, should be considered in the setting of AVM hemorrhage with paradoxical motor impairment and can be identified through MR imaging.

## Abbreviations

AVM: Arteriovenous malformation
ICH: Intracerebral hemorrhage
SDH: Subdural hematoma
CT: Computed tomography
MR: Magnetic resonance
MRI: Magnetic resonance imaging.
